# *De novo* assembly and functional annotation of *Citrus aurantifolia* transcriptome from *Candidatus* Liberibacter asiaticus infected and non-infected trees

**DOI:** 10.1016/j.dib.2020.105198

**Published:** 2020-01-25

**Authors:** Ángela Paulina Arce-Leal, Rocío Bautista, Edgar A. Rodríguez-Negrete, Miguel Ángel Manzanilla-Ramírez, José Joaquín Velázquez-Monreal, Jesús Méndez-Lozano, Eduardo R. Bejarano, Araceli G. Castillo, M. Gonzalo Claros, Norma Elena Leyva-López

**Affiliations:** a Instituto Politécnico Nacional, CIIDIR-Unidad Sinaloa, Departamento de Biotecnología Agrícola, Mexico; b Plataforma Andaluza de Bioinformática, Universidad de Málaga, Malaga, Spain; c CONACyT, Instituto Politécnico Nacional, CIIDIR-Unidad Sinaloa, Departamento de Biotecnología Agrícola, Mexico; d Campo Experimental Tecomán-INIFAP, Carretera Colima-Manzanillo km. 35, Tecomán, Colima, Mexico; e Instituto de Hortofruticultura Subtropical y Mediterránea La Mayora (IHSM-UMA-CSIC), Área de Genética, Facultad de Ciencias, Universidad de Málaga, Málaga, Spain; f Departamento de Biología Molecular y Bioquímica, Universidad de Málaga, Malaga, Spain

**Keywords:** *Citrus aurantifolia*, Huanglongbing, *Candidatus* Liberibacter asiaticus, Infection early and late stages, RNA-Seq, Transcriptome assembly

## Abstract

Mexican lime (*Citrus aurantifolia*) belongs to the Rutaceae family and nowadays is one of the major commercial citrus crops in different countries. In Mexico, Mexican lime production is impaired by Huanglongbing (HLB) disease associated to *Candidatus* Liberibacter asiaticus (CLas) bacteria. To date, transcriptomic studies of CLas-Citrus interaction, have been performed mainly in sweet citrus models at symptomatic (early) stage where pleiotropic responses could mask important, pathogen-driven host modulation as well as, host antibacterial responses. Additionally, well-assembled reference transcriptomes for acid limes including *C. aurantifolia* are not available. The development of improved transcriptomic resources for CLas-citrus pathosystem, including both asymptomatic (early) and symptomatic (late) stages, could accelerate the understanding of the disease. Here, we provide the first transcriptomic analysis from healthy and HLB-infected *C. aurantifolia* leaves at both asymptomatic and symptomatic stages, using a RNA-seq approach in the Illumina NexSeq500 platform. The construction of the assembled transcriptome was conducted using the predesigned workflow Transflow and a total of 41,522 tentative transcripts (TTs) obtained. These *C. aurantifolia* TTs were functionally annotated using TAIR10 and UniProtKB databases. All raw reads were deposited in the NCBI SRA with accession numbers SRR10353556, SRR10353558, SRR10353560 and SRR10353562. Overall, this dataset adds new transcriptomic valuable tools for future breeding programs, will allow the design of novel diagnostic molecular markers, and will be an essential tool for studying the HLB disease.

**Table:** Specifications Table

Subject	Plant Biology
Specific subject area	Transcriptomics
Type of data	RNA Sequencing Data
How data were acquired	Illumina NexSeq500 sequencing platform
Data format	Raw and analyzed
Parameters for data collection	Leaves of *C. aurantifolia* were collected from shadow-greenhouse at the Experimental Station Tecoman-INIFAP, Tecoman, Colima, Mexico. The samples were taken at four conditions: asymptomatic CLas-infected plants (early stage of HLB disease), symptomatic CLas-infected plants (late stage of HLB disease), and mock-inoculated control plants at both time points, early and late stages of the HLB infection.
Description of data collection	The RNA was sequenced separately (2 × 150 bp) on an Illumina NexSeq500 instrument MID-Output in Langebio-CINVESTAV, Mexico.
Data source location	Instituto Politécnico Nacional, CIIDIR-Unidad Sinaloa. Guasave, Sinaloa, Mexico (25°32′42.7″N 108°28′53.4″W)
Data accessibility	NCBI SRA accession numbers of SRR10353562, https://www.ncbi.nlm.nih.gov/sra/SRR10353562, SRR10353560 https://www.ncbi.nlm.nih.gov/sra/SRR10353560, SRR10353558 https://www.ncbi.nlm.nih.gov/sra/SRR10353558, SRR10353556 https://www.ncbi.nlm.nih.gov/sra/SRR10353556 for raw reads; https://data.mendeley.com/datasets/99tbnvjhsh/1 for the assembled and annotated transcriptome.

**Table:** 

**Value of the Data** • This data provides the first transcriptome of *Citrus aurantifolia* that merges the information from CLas-infected and non-infected leaves tissue • The *de novo* assembled transcriptome is useful as a reference transcriptome to other scientists working in the prediction and functional annotation of differentially expressed genes in Mexican lime and other acid limes. • The RNA-seq dataset is available as raw sequence reads that can be further processed and analyzed by scientists. • This data could be useful for citrus breeding programs and the designing of novel diagnostic tools.

## 1 Data

**Fig. 1:**
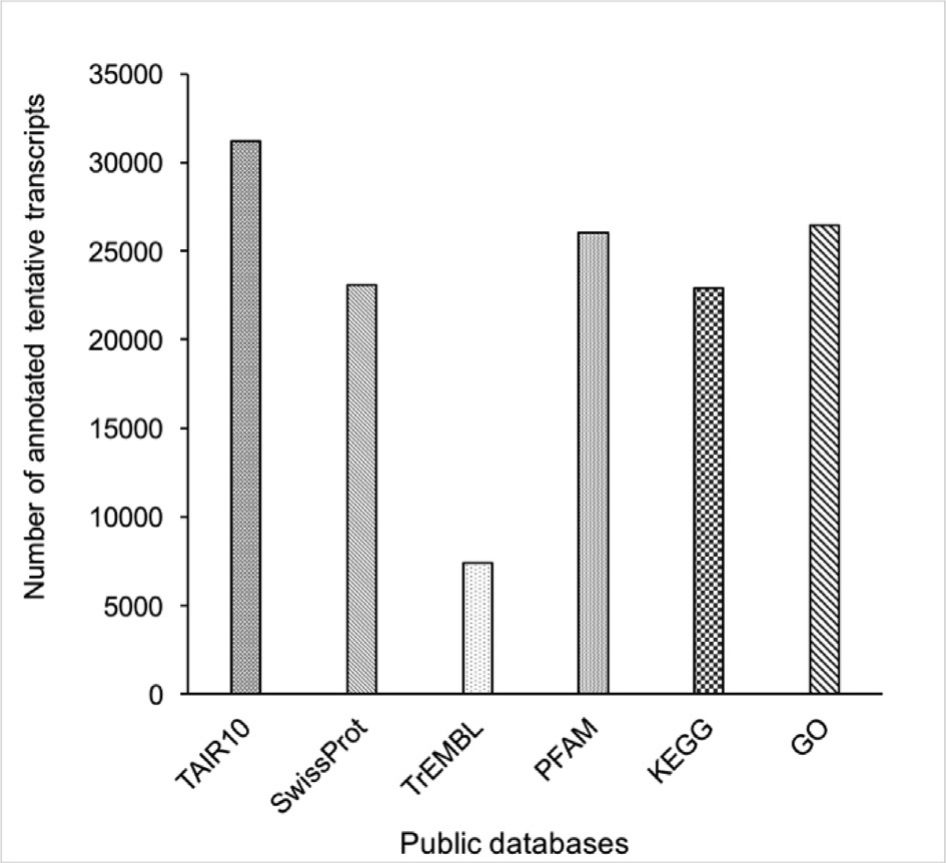
Number of annotated tentative transcripts of the *C. aurantifolia* transcriptome using different public databases.

**Fig. 2:**
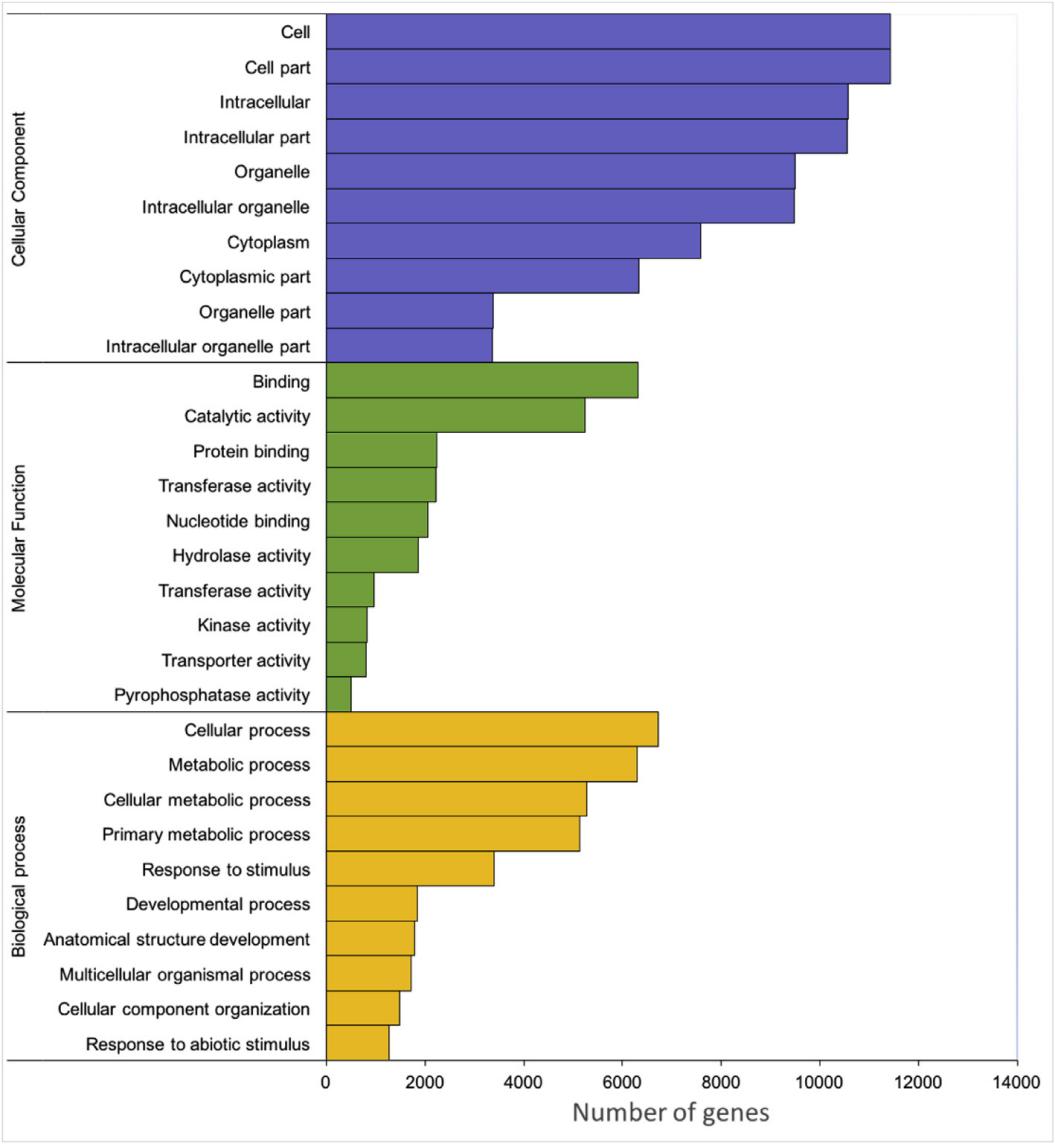
GO category distribution of AgriGO annotated *C. aurantifolia* Tentative Transcripts.

The transcriptome assembly and its annotation for *Citrus aurantifolia* leaves were generated from RNA samples including four conditions: asymptomatic CLas-infected plants (early stage of HLB disease, 8 weeks post inoculation, wpi), symptomatic CLas-infected plants (late stage of HLB disease, 16 wpi) and mock-inoculated control plants at both time points, 8 and 16 wpi. The four cDNA libraries were sequenced using the Illumina NexSeq500 platform, resulting in a total of 110, 572, 474 raw reads (Table 1). After removal of low-quality reads, eight different *de novo* assemblies were generated. Based on of the structural annotation statistics of the full set of tentative transcripts, the best *de novo* assembled transcriptome is then presented in Table 2. This assembly was functionally annotated by TAIR 10 database, and with the plant division for UniProtKB (Fig. 1) and then Gene Ontology (GO) identity was assigned (Fig. 2).

**Table 1:** Summary of raw data and clean reads for each accession. Raw Reads: reads from next-generation sequencer. Clean Reads: high quality reads after eliminating contaminations and adaptors. Clean Reads%: the percentage of clean reads.

Library	Combined number of reads (paired-end)	
	Raw reads	Clean reads
L8wpiHLB+1	28,409,266	23,860,630
L8wpiHLB-3	29,239,848	24,967,772
L16wpiHLB+5	26,237,006	22,153,278
L16wpiHLB-7	26,686,354	23,111,948
**Total reads**	110,572,474	90,093,628

**Table 2:** Structural annotation statistics of the full set of tentative transcripts Oases *K-*35/55 CD-Hit, the best *de novo* assembled transcriptome.

Property		Tentative transcripts	Percentage
Total tentative transcripts		41,680	
Tentative transcripts		41,522	100.00%
Tentative transcripts >500pb		35,735	86.06%
With orthologue		34,123	82.18%
Different orthologue IDs		16,426	48.14%
Complete transcripts		26,614	77.99%
Different complete transcripts		13,294	38.96%
ncRNA		405	0.98%
Without orthologue		6994	16.84%
Coding (all)		1438	20.56%
Coding > 200bp		1438	20.56%
Coding > 500bp		1084	15.50%
Unknown (all)		5556	79.44%
Artifacts		158	0.38%
**Status**			
Complete	Sure	19,013	45.79%
	Putative	7601	18.31%
C-terminus	Sure	781	1.88%
	Putative	2769	6.67%
N-terminus	Sure	1967	4.74%
	Putative	607	1.46%
Internal		1385	3.34%
Coding	Sure	296	0.71%
	Putative	1142	2.75%
ncRNA		405	0.98%
Unknown		5556	13.38%

Raw RNA-seq reads and the *de novo* transcriptome assembly can be accessed at the NCBI with the following accession numbers: SRR10353562 for the RNA-Seq of asymptomatic Mexican lime infected with CLas (8 wpi), SRR10353558 for the RNA-Seq of symptomatic Mexican lime infected with CLas (16 wpi) and SRR10353560 and SRR10353556 for the RNA-Seq of mock-inoculated Mexican lime (negative control plants) at 8 and 16 wpi, respectively.

## 2 Experimental design, materials, and methods

### 2.1 Plant materials

Mexican lime (*C. aurantifolia*) plants on alemow (*C. macrophylla*) rootstock were kept in a pathogen free shadow-greenhouse at Experimental Station Tecoman-INIFAP, Tecoman, Colima, Mexico. Forty-five 9 months-old Mexican lime (*C. aurantifolia*) plants were CLas-inoculated by grafting with budwood from HLB-infected Mexican lime trees as inoculum source, and other fifteen plants were inoculated with budwood from healthy Mexican lime plants as negative control plants (mock-inoculated). Following inoculation, plants were kept in a shadow-greenhouse and fertilized if necessary. Foliar tissue including complete leaves and petioles (8 leaves) were collected from individual plants, at 8 and 16-weeks post inoculation (wpi) (asymptomatic/early, and symptomatic/late stages, respectively). For each sampled plant, 4 complete leaves were grinded with mortar and pestle in liquid nitrogen (for RNA-Seq analysis). The remaining 4 leaves were used for dissection of central midribs and petioles (tissue where bacteria is mainly located) for bacteria detection, and liquid nitrogen grinded. Finally, the tissue powder was stored at −80 °C prior to use. To quantify the CLas bacterial titer in grafting inoculated *C. aurantifolia* plants, an absolute quantitative PCR (qPCR) assay was performed [1]. Total DNA was extracted from the powdered tissue from midribs and petioles (about 200 mg) from individual plants, using a previously described CTAB protocol [2,3]. Bacterial titer quantification of the forty-five CLas-infected plants was carried out at both 8 and 16 wpi. Five CLas-infected plants with a similar bacterial titer at each time point (2.2 ± 1.1 × 10^2^ bacterial cells/100 ng of total DNA at 8 wpi and 1.2 ± 0.8 × 10^4^ bacterial cells/100 ng of total DNA at 16 wpi) and the corresponding five mock-inoculated control plants (at 8 and 16 wpi), were selected as tissue source for the RNA-seq analysis. The grinded leaf tissue from the five plants selected for each condition and time point, were pooled for the RNA extraction.

### 2.2 RNA sequencing, *de novo* assembly and transcript annotation

Total RNA was isolated according to TRIzol® protocol (Sigma-Aldrich) from tissue of complete leaves. The yield and quality of the RNA was verified by assessing the A_280_/A_260_ ratio by Nanodrop 2000 Spectrophotometer, and RNA integrity was determined using a 2100 Bioanalyzer RNA Nanochip (Agilent, CA, USA). Total RNA from selected plants for each condition was pooled in equimolar ratio to construct each cDNA libraries. The cDNA libraries with fragments ≈500 base pairs (bp) were constructed according to the manufacturer's instructions using the TruSeq Stranded mRNA Sample Preparation kit (Illumina, San Diego, CA), and sequenced separately (2 × 150 bp) on an Illumina NexSeq500 instrument MID-Output by Langebio-CINVESTAV, Irapuato facilities (Mexico). A total of 110, 572, 474 (more than 25 million reads for each library) raw reads were generated. Quality of raw reads was assessed with FastQC tool with default parameters and then pre-processed to remove adapter and contaminant sequences and low quality reads using SeqTrimNext [4]. After removal of low-quality reads, 90, 093, 628 clean reads (i.e. 85 % of raw reads) were then assembled to generate eight *C. aurantifolia* transcriptomes using TransFlow [5] which is a modular assembling framework that combines different assembling strategies to finally select the most accurate *de novo* transcriptome. Assembling strategies of clean reads was based on two assemblers based on Bruijn algorithms: Oases [6] and SOAPdenovo [7] using *K*-*mer* parameters of 35, 55, and combined. Additionally, to reduce redundancy, the resulting contigs obtained with Oases *K*-*mers* 35/55 and SOAPdenovo *K*-*mers* 35/55 were clustered with CD-HIT [8] and then reconciled with Minimus [9] using the default parameters. Completeness and quality of the eight assemblies were analyzed as described for TransFlow [5] to identify the best transcriptome assembly based on principal component analysis (PCA). The best assembling strategies correspond to the ones having the shortest distance to the *A. thaliana* (TAIR10) reference transcriptome. Finally, structural annotation of the complete set of TTs from Oases *K-mers* 35/55 CD-Hit assembling was obtained using Full-LengtherNext [5], preferentially annotating with *A. thaliana* proteome from TAIR 10 database, and then with the plant division of UniProtKB. The best transcriptome consisted of 41,522 Tentative Transcripts (TTs). The protein-transcript orthology was assessed using Full-LengtherNext (5). Gene ontology (GO) enrichment was analyzed by AgriGO version 2.0 [10]. Enriched GO terms were selected using Singular Enrichment Analysis (SEA) with the *A. thaliana* reference genome as background (TAIR10_2017). The over-represented terms in the three categories biological process, cellular component, and molecular function were filtered by statistical information using an FDR-adjusted p-value of ≤0.05.
